# *PePIF1*, a P-lineage of *PIF*-like transposable element identified in protocorm-like bodies of *Phalaenopsis* orchids

**DOI:** 10.1186/s12864-018-5420-4

**Published:** 2019-01-09

**Authors:** Chia-Chi Hsu, Pei-Han Lai, Tien-Chih Chen, Wen-Chieh Tsai, Jui-Lin Hsu, Yu-Yun Hsiao, Wen-Luan Wu, Ching-Hsiu Tsai, Wen-Huei Chen, Hong-Hwa Chen

**Affiliations:** 1 Department of Life Sciences, National Cheng Kung University, Tainan, Taiwan; 2 Institute of Tropical Plant Sciences, National Chung Hsing University, Tainan, Taiwan; 3 Orchid Research and Development Center, National Cheng Kung University, Tainan, Taiwan; 4 Graduate Institute of Biotechnology, National Chung Hsing University, Taichung, Taiwan

**Keywords:** Micropropagation, *PePIF1*, *Phalaenopsis*, Protocorm-like bodies, Somaclonal variation, Transposable elements, Transposon display

## Abstract

**Background:**

Orchids produce a colorless protocorm by symbiosis with fungi upon seed germination. For mass production of orchids, the prevailing approaches are both generation of protocorm-like bodies (PLBs) from callus and multiplication of adventitious buds on inflorescence. However, somaclonal variations occur during micropropagation.

**Results:**

We isolated the two most expressed transposable elements belonging to *P Instability Factor* (*PIF*)-like transposons. Among them, a potential autonomous element was identified by similarity analysis against the whole-genome sequence of *Phalaenopsis equestris* and named *PePIF1*. It contains a 19-bp terminal inverted repeat flanked by a 3-bp target site duplication and two coding regions encoding ORF1- and transposase-like proteins. Phylogenetic analysis revealed that *PePIF1* belongs to a new P-lineage of *PIF*. Furthermore, two distinct families, *PePIF1a* and *PePIF1b*, with 29 and 37 putative autonomous elements, respectively, were isolated, along with more than 3000 non-autonomous and miniature inverted-repeat transposable element (MITE)-like elements. Among them, 828 *PePIF1*-related elements were inserted in 771 predicted genes. Intriguingly, *PePIF1* was transposed in the somaclonal variants of *Phalaenopsis* cultivars, as revealed by transposon display, and the newly inserted genes were identified and sequenced.

**Conclusion:**

A *PIF*-like element, *PePIF1*, was identified in the *Phalaenopsis* genome and actively transposed during micropropagation. With the identification of *PePIF1*, we have more understanding of the *Phalaenopsis* genome structure and somaclonal variations during micropropagation for use in orchid breeding and production.

**Electronic supplementary material:**

The online version of this article (10.1186/s12864-018-5420-4) contains supplementary material, which is available to authorized users.

## Background

Orchidaceae, containing more than 25,000 species, is one of the largest angiosperm families and is distributed in most land areas. The capsules contain hundreds of thousands of dust-like seeds that germinate and produce colorless protocorms by symbiosis with fungi in nature. The most popular orchid, the genus *Phalaenopsis*, comprises approximately 66 species [1], and more than 30,000 hybrid cultivars are registered in the Royal Horticultural Society [2]. *P. equestris* is a model orchid plant for genomic study that relies on the groundwork of basic genomics information [3]. OrchidBase (http://orchidbase.itps.ncku.edu.tw) has been established with collected transcriptome libraries from 11 *Phalaenopsis* orchids [4, 5], and Orchidstra (http://orchidstra.abrc.sinica.edu.tw) has been constructed for tissue-specific expression profiles in *P. aphrodite* [6]. The whole-genome sequence of *P. equestris* was published [3] and is available in OrchidBase 3.0.

In the orchid nursery, micropropagation is performed to maintain an elite orchid variety derived from a cross between two parents with desirable traits. Multiplication of adventitious buds or induction of protocorm-like bodies (PLBs) from callus are the two major approaches. Mass propagation produces plantlets with uniform growth and flowering time and eases the management of the orchid nursery. However, among the thousands of plantlets, some mutant phenotypes due to somaclonal variation are found during the vegetative or reproductive stage [7]. Usually, the occurrence rates of somaclonal variation are higher with induction of PLBs than multiplication of adventitious buds and continuous multiplication of consecutive generations of PLBs. Thus, orchid growers usually use induction of PLBs or multiplication of adventitious buds with fewer than three generations to avoid a high rate of somaclonal variation.

Somaclonal variation studies have focused on the activation of transposable elements (TEs), DNA methylation status, and histone modifications in several crops, such as oil palm, rice, and tobacco [8–13]. TEs are defined as DNA fragments that can transpose to new locations of chromosomes and are responsible for chromosomal rearrangements [14–16], fragmental gene movements [17, 18], and the evolution of gene regulation and function [19, 20]. Most main TE groups are ancient and are present in all kingdoms, and thousands to tens of thousands of TE families are found in plants [18, 21–23]. Two major classes of TEs were classified and distinguished by their transposition intermediate: class-I RNA retrotransposons or class-II DNA transposons. Class-I retrotransposons use the transposition mechanism of “copy-and-paste”, whereas class-II transposons use “cut-and-paste” [24]. However, miniature inverted TEs (MITEs) are considered non-autonomous DNA transposons but have multitudinous copy numbers within a genome [18, 24–26]. MITEs lack coding sequences, so their transposition is considered to be activated by autonomous elements of class-II DNA transposons, which share similar terminal inverted repeats (TIRs) with MITEs.

Two major MITEs have been identified in plants: *Tourist*-like, with 3-bp target-site duplications (TSDs), usually TTA/TAA, and *Stowaway*-like, with 2-bp TSDs, usually TA [22]. Two pairs of active *Tourist*-like MITE families with their related autonomous class-II DNA transposons have been identified: a 364-bp *miniature P Instability Factor* (*mPIF*) with transposase (TPase)-encoding elements, *PIF* in maize [27, 28], and a 430-bp *mPing* with autonomous *Ping* and *Pong* in rice [29–31]. *PIF* and *Pong* elements share several features, including conserved amino acid sequences in their open reading frames (ORFs) and nucleotide sequence homology in their TIRs and TSDs [29, 32].

The autonomous *PIF* element contains two ORFs: ORF1, coding for a Myb/SANT-containing protein with unknown function, and TPase, with a catalytic DDE motif for transposition [33]. *mPIF* shares features with *PIF* elements, including identical 14-bp TIRs, similar sub-terminal sequences, and an extended 9-bp target site preference [28]. Several *PIF*-like elements that have been identified with no analysis of their transposition activities include an autonomous 4.4-kb *DcMaster-a* and a 2.5-kb *DcMaster1* in carrot (*Daucus carota* L.), a 5.14-kb *MtMaster-a* in *Medicago truncatula*, and a full-length 5.9-kb *PpPIF-1* in bamboo (*Phyllostachys pubescens*) [34–37]. Recently, the genome-wide identification of *PIF*-like elements was analyzed in 21 species of Triticeae genera by using PCR-based approaches for conserved TPase sequences in genomes and transcriptomes [32, 37].

Previously, a homemade Orchid Oligo Array (4 × 44 K) was developed and contained 14,732 gene-specific oligonucleotides (45–60 mers in length) derived from 84,617 unigenes in OrchidBase [38]. The Orchid Array was used to analyze the gene expression profiles for the consecutive PLB generations of the somaclonal variant *P.* Brother Spring Dancer ‘KHM487’, which has crystal-like PLBs [38]. In this study, we successfully identified an active TE, *PePIF1*, in a *Phalaenopsis* genome by combining microarray data with in silico analysis and transposon display experiments.

## Results

### Transcriptional activation of *Phalaenopsis* TEs during PLB micropropagation

**Fig. 1:**
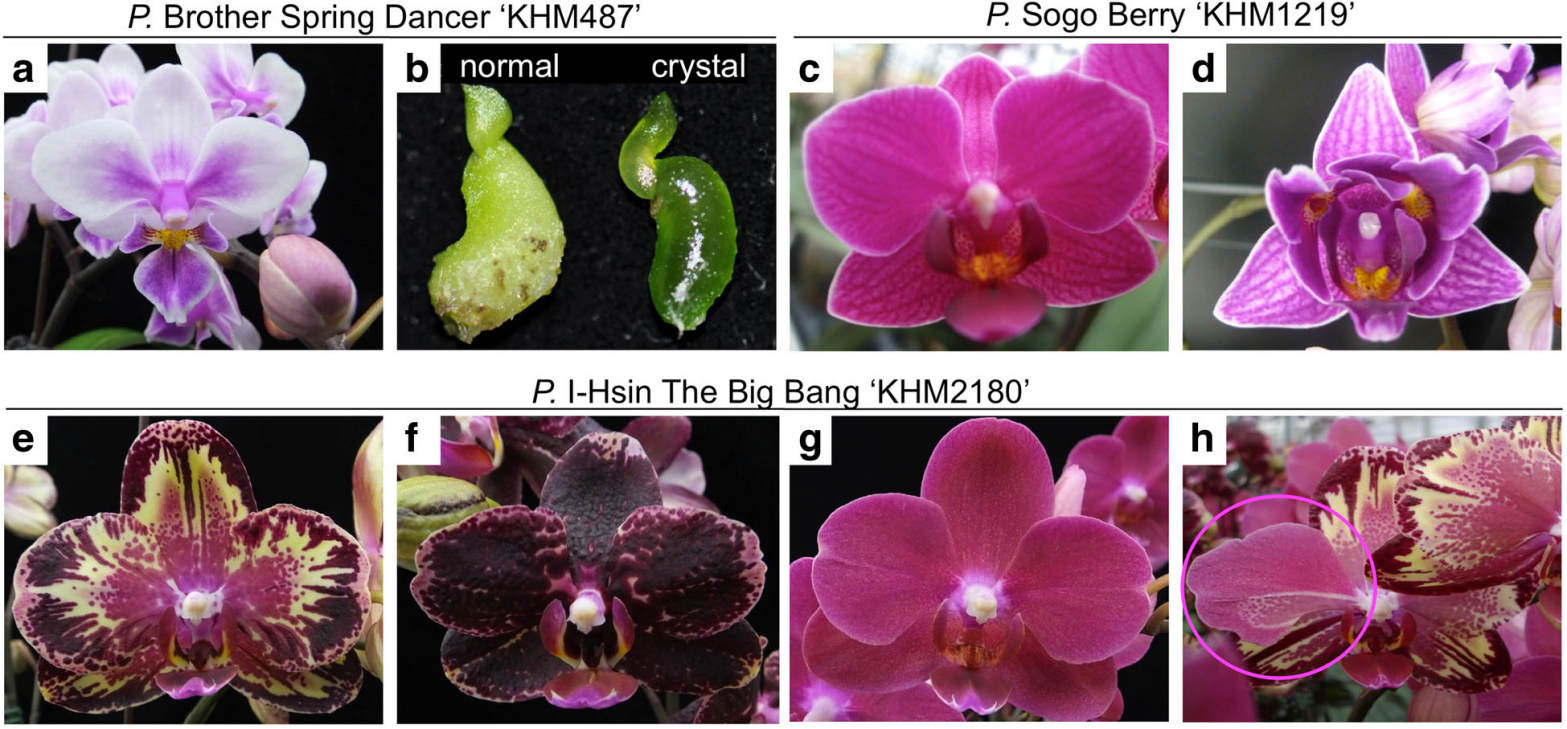
Three pairs of normal and somaclonal variants of *Phalaenopsis*, including *P.* Brother Spring Dancer ‘KHM487’ (KHM487, **a** and **b**), *P.* Sogo Berry ‘KHM1219’ (KHM1219, **c** and **d**), and *P.* I-Hsin The Big Bang ‘KHM2180’ (KHM2180, **e**-**h**). These somaclonal variants contain different phenotypes: KHM487 with the crystal-like protocorm-like bodies (PLBs) (**b**), KHM1219 having peloric flowers (**d**) and different flower color patterns for KHM2180 (**e**-**h**)

**Fig. 2:**
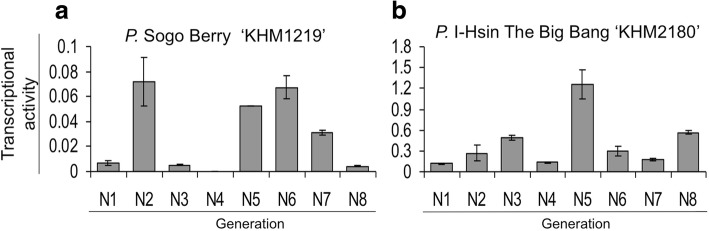
Transcription activity of *EFCP_7972* during tissue culture of KHM1219 (**a**) and KHM2180 (**b**). Data are mean ± SEM from experiments performed in triplicate

We searched for enhanced expression of transposon- or retrotransposon-like elements from microarray data for various PLB generations of KHM487 (Fig. 1a, b). It is plausible that an active TE with enhanced transcription level during micropropagation may cause somaclonal variations. Two unigenes, *EICPS_047* and *EFCP_7972*, annotated as *PIF*-like TPases with a significant increase in expression in the 6th PLB generation (Table 1) were chosen as the TE candidates in this study. Quantitative real-time PCR was performed to confirm the increased transcription of the TE candidates with RNA from consecutive PLB generations (N1-N8) of both *P.* Sogo Berry ‘KHM1219’ and *P.* I-Hsin The Big Bang ‘KHM2180’ (abbreviated as KHM1219 and KHM2180, respectively), which feature peloric flowers and several floral color patterns in the somaclonal variants, respectively (Fig. 1c-h). The TE candidates expressed in the eight consecutive generations to various extents (Fig. 2; Additional file 1: Figure S1). Among them, *EFCP_7972* highly expressed in the 5th PLB generation (N5) of KHM1219 and KHM2180 and in the 2nd generation (N2) of KHM1219 (Fig. 2). These results suggest that the TPase was expressed during *Phalaenopsis* tissue culture and reached high expression level at least in one PLB generation. However, the differential transcription activities among PLB generations reflected that not all PLBs showed *EFCP_7972* expression in every PLB generation during tissue culture, which concerns the silencing effects on TEs in plant genome.

**Table 1:** Expression patterns of putative transposable elements at various protocorm-like body (PLB) generations by microarray analysis of somaclonal variations of *P.* Brother Spring Dancer ‘KHM487’

Gene	Annotation	KHM487		
		4th / original	5th / original	6th / original
*Consensusfrom contig1*	retrotransposon	ND^a^	ND	0.33^b^
*FCTEVCPM_103*	Putative retrotransposon polyprotein	ND	ND	0.47
*FEVCPM_022*	PIF-like transposase	ND	ND	0.50
*FEVCPM_036*	*Ty1*/*Copia* retrotransposon	ND	ND	2.10
*EICPS_047* ^c^	PIF-like transposase	ND	ND	2.72
*EFCP_7972* ^c^	PIF-like transposase	ND	ND	3.52
*FPEAECP015*	Transposon protein	0.39	ND	ND

^a^: ND, undetected

^b^: Relative expression levels in microarray analysis

^c^: Genes with highly increased expression patterns on the 6th tissue culture generation derived from *P.* Brother Spring Dancer ‘KHM487’ and were analyzed in this paper

### Genome structure analysis reveals EFCP_7972 as a potential autonomous element

**Fig. 3:**
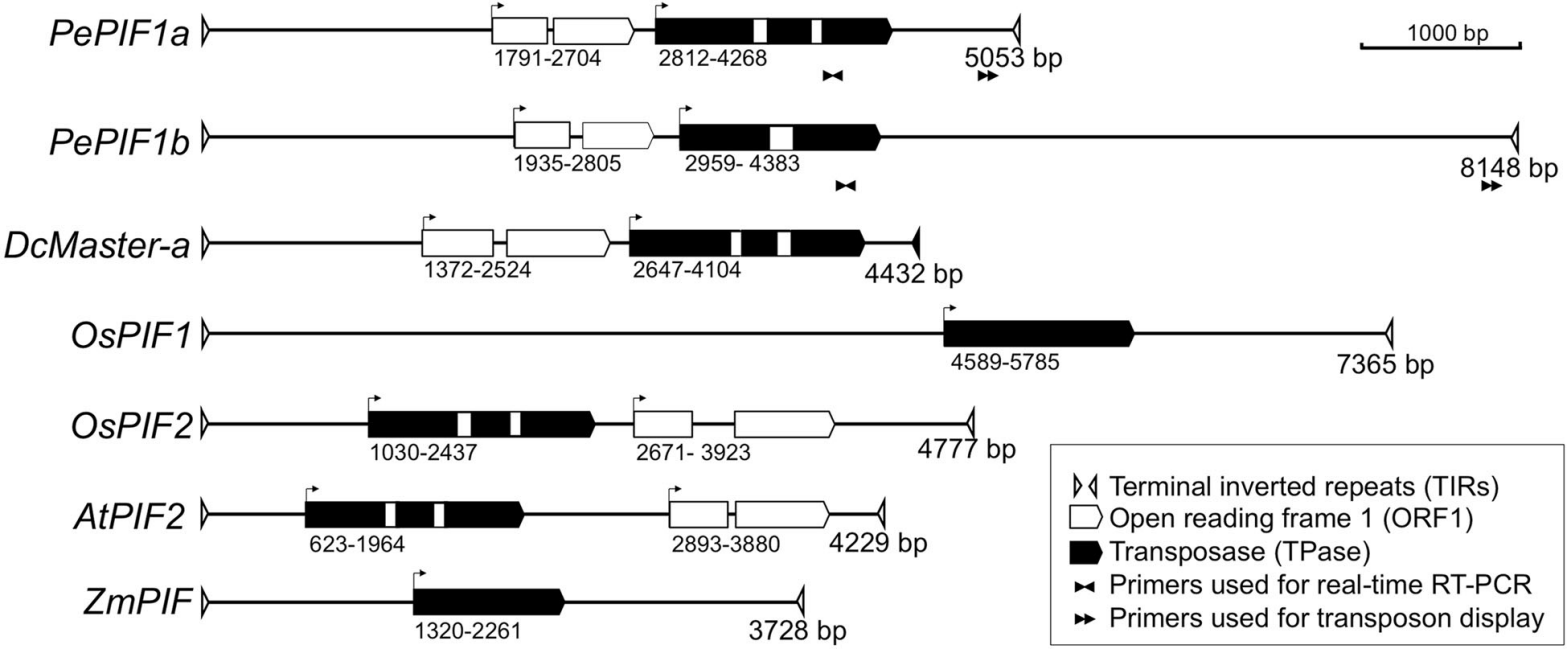
The transposable element (TE) structures of *PePIF1* in the *Phalaenopsis* genome and other *PIF*-like elements. The triangles indicate the identified terminal inverted repeats (TIRs). Black and hatched rectangles represent the open reading frames for TPase and ORF1, respectively, and blocked rectangles indicate the presence of introns

The TE candidates were mapped to the whole-genome sequence of *P. equestris* [3] to identify their genome structures in *Phalaenopsis*. Within the 1.1-Gb assembled *P. equestris* genome, *EICPS_047* was detected with one hit and *EFCP_7972* was detected with 369 hits (Additional file 2: Table S1). The top hit in the BLAST results for *P. equestris* was used to identify the genome structures for each TE candidate. *EFCP_7972* appeared as an intact element with the presence of TIRs in both ends (Fig. 3), but *EICPS_047* was not surrounded by repeat sequences. We then focused on *EFCP_7972* for the following analysis.

*EFCP_7972* was annotated as a *PIF*-like transposon and mapped in Scaffold000759 (Additional file 2: Table S1), containing a 19-bp TIR with the sequence GGGYCYGTTTGGGGCAGCT (Y represents C/T) and flanked by the 3-bp TSD, TTA (Fig. 3; Table 2). Two ORFs with a head-to-tail direction were identified within the TIRs, coding for an ORF1- and a TPase-like protein, respectively (Fig. 3). This sequence was then renamed *P. equestris PIF1* (*PePIF1*).

**Table 2:** Terminal inverted repeats (TIRs) and target-site duplication (TSDs) of *PePIF1* and other *PIF/Harbinger*-like transposable elements

Element	Full length (bp)	TIR length (bp)	TIR sequence	TSD	Length of ORF1 (a.a.)	Length of TPase (a.a.)	Reference	Accession no.
*PePIF1*	5053	19	GGGYCYGTTTGGGGCAGCT	TTA	272	427	This study	
*DcMaster-a*	4432	22	GKGYCTGTTTGGSRTTGCKGTT	3 bp	353	425	Grzebelus et al., 2006	AC144478
*AtPIF2*	4229	20	GGKGGTGTTATTGGTTAGTG	TTA	303	399	Zhang et al., 2001	AF007271
*OsPIF1*	7365	15	GGCCTYGTTTGGCTG	TTA	-^a^	398	Zhang et al., 2001	AC025098
*OsPIF2*	4777	14	GGGGTTGTTTGGTT	ATA	303	411	Zhang et al., 2001	AP001111
*ZmPIFa*	3728	14	GGGCCCGTTTGTTT	TTA	-^a^	298	Zhang et al., 2001	AF412282
*Harbinger*	1530	25	GGTCCTGTTTGTTTGTCCATTTGGA	3 bp	-^a^	-^a^	Kapitonov and Jurka, 1999	JX556412

^a^: ORF1 not found

ORF1 of *PePIF1*, containing two exons encoding a 272-amino acid protein, shows 36% identity to the ORF1 of *DcMaster-a* [34] (Fig. 3; Table 2) and contains a conserved MYB/SANT motif with uncharacterized function (Additional file 3: Figure S2a). ORF2 is a 427-aa TPase-like protein encoded by three exons (Fig. 3) and a catalytic DDE motif within the conserved residues; N2, N3 and C1 regions; and a helix-turn-helix (HTH) domain involved in DNA binding (Additional file 3: Figure S2b, underlined regions) [33].

### *PePIF1* belongs to a new P lineage of *PIF*-like elements separate from other plants

**Fig. 4:**
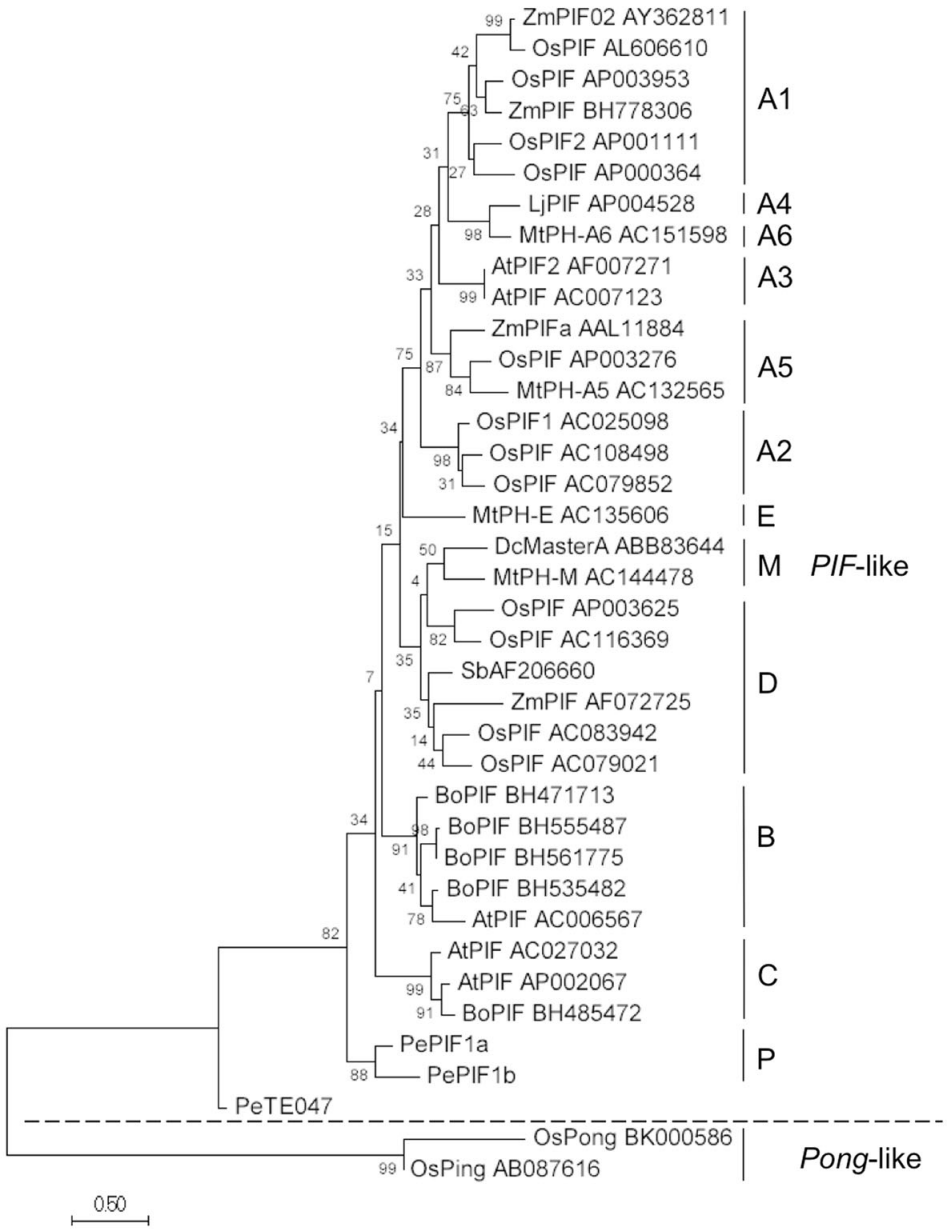
Phylogenetic analysis of TPase from *PIF*- and *Pong*-like elements. The other *PIF*-like elements are named according to the species initials followed by their GenBank accession number. The plant names are At: *Arabidopsis thaliana*, Bo: *Brassica oleracea*, Dc: *Daucus carota*, Lj: *Lotus japonicus*, Mt.: *Medicago truncatula*, Os: *Oryza sativa*, Pe: *Phalaenopsis equestris*, Sb: *Sorghum bicolor*, Zm: *Zea mays*

A phylogenetic tree of *PePIF1* was constructed with the conserved catalytic DDE motif of the *PIF*-like TPase and compared to the *Pong*-like TPase by the maximum likelihood method. *PePIF1* was separate from the *Pong*-like elements and belongs to a *PIF*-like element (Fig. 4). *PePIF1* was well separated from the previous identified lineages, including A1~A6, B, C, D, E and M, and thus grouped into a whole new lineage, named P lineage, for *Phalaenopsis* (Fig. 4).

To investigate whether *PePIF1* was amplified within the *Phalaenopsis* genome, we searched for other copies of *PePIF1* based on the presence of TIRs, TPase and ORF1 within the whole-genome sequence of *P. equestris*. A total of 66 putative autonomous elements was isolated based on the presence of the coding sequences of TPase and ORF1 within two TIRs and could be divided into *PePIF1a* and *PePIF1b* with 29 and 37 elements, respectively (Fig. 4; Table 3; Additional file 4: Table S2). *PePIF1a* and *PePIF1b* shared the same 19-bp TIR and 3-bp TSD but contained only 51.6 and 37.4% amino acid identity with TPase and ORF1, respectively. Thus, *PePIF1a* and *PePIF1b* were considered two distinct families according to the 80–80-80 rule [24]. The *PePIF1a* family contains the *EFCP_7972* sequence located on Scaffold000759, with its TPase encoded by three exons, whereas *PePIF1b* was identified in Scaffold000402, with its TPase encoded by two exons (Table 3). Most of these predicted autonomous elements showed 5~6- and 6~8-kp sequences for *PePIF1a* and *PePIF1b*, respectively (Table 3), and the shorter or longer elements might result from the deletion or insertion events, respectively.

**Table 3:**
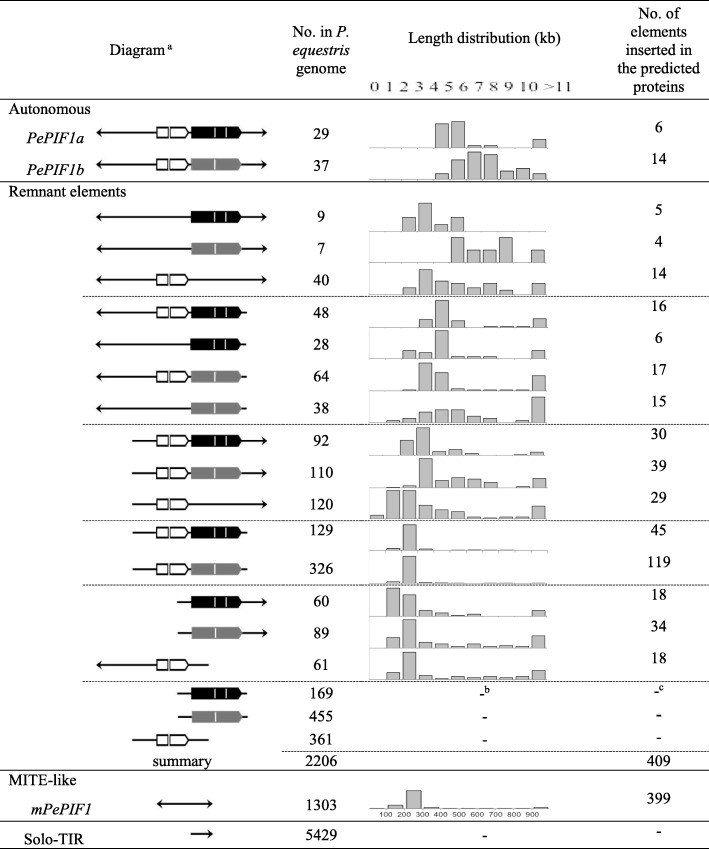
Number of autonomous and defective elements of *PePIF1* in the whole-genome sequence of *P. equestris*. The presence of TIR, ORF1, TPaseA, TPaseB are shown with an arrowhead, white, gray, and black frames, respectively

^a^: The diagrams for the TEs are shown as arrows: TIR, white frames: ORF1, black frames: TPaseA and gray frames: TPaseB

^b^: The percentage of length distribution was not analyzed for the TPase, ORF1 or TIRs alone

^c^: The number of elements inserted in the predicted proteins were not analyzed for the TPase, ORF1 or TIRs alone

In addition to the intact autonomous elements, abundant defective copies that lost their TIRs, coding sequences of TPase or ORF1 were detected (Table 3). The length distributions of these remnant elements decreased in accordance with the defective TIR or coding sequences. For example, the remnant elements without ORF1 were 3~4-kb and 5~6-kb long from *PePIF1a* and *PePIF1b*, respectively (Table 3); 3~5-kb long for defective elements with the loss of one TIR, and 2~3-kb long with the loss of two TIRs (Table 3). Further deletion of one TIR and one coding sequence resulted in the shortest elements, only with 1~2-kb sequences (Table 3). In addition, 1303 copies of MITE-like elements with both TIRs were identified without any coding sequences, most being 200 to 300 bp long (Table 3). In total, more than 3000 copies of *PePIF1* were present in the *Phalaenopsis* genome, yet most were defective copies or MITE-like elements.

### Abundant *PePIF1* insertions in the coding sequences of the *Phalaenopsis* genome

In analyzing the *P. equestris* genome, we noted a high proportion of TEs located in gene regions [3]. To investigate whether the *PIF*-like elements were inserted in the coding sequences, we analyzed flanking sequences of *PePIF1* in the *Phalaenopsis* genome. A total of 828 *PePIF1*-related elements inserted into 771 predicted proteins were identified in the genome sequence of *P. equestris* (Table 3). A rough view for 100 predicted genes with *PePIF1* insertions, there were 76 intronic regions and 24 genes containing partial coding sequences of *PePIF1*, concomitant with our previous observation that *P. equestris* genome contains abundant intronic TE, and that alters the expression of coding genes [3]. Similarly, intronic insertion of *Alu* elements causes alternative splicing [39]. Among them were 6 and 14 putative autonomous elements of *PePIF1a* and *PePIF1b* families, respectively; 409 defective elements of *PePIF1*; and 399 MITEs identified as insertions within the predicted gene regions (Table 3). In addition, within the 771 predicted proteins in the *P. equestris* genome, 594 predicted genes encode non-transposon genes, 6 encode transposable elements, and the other 171 annotated to uncharacterized proteins.

### Transpositional activity of *PePIF1* during consecutive PLBs analyzed by transposon display

**Fig. 5:**
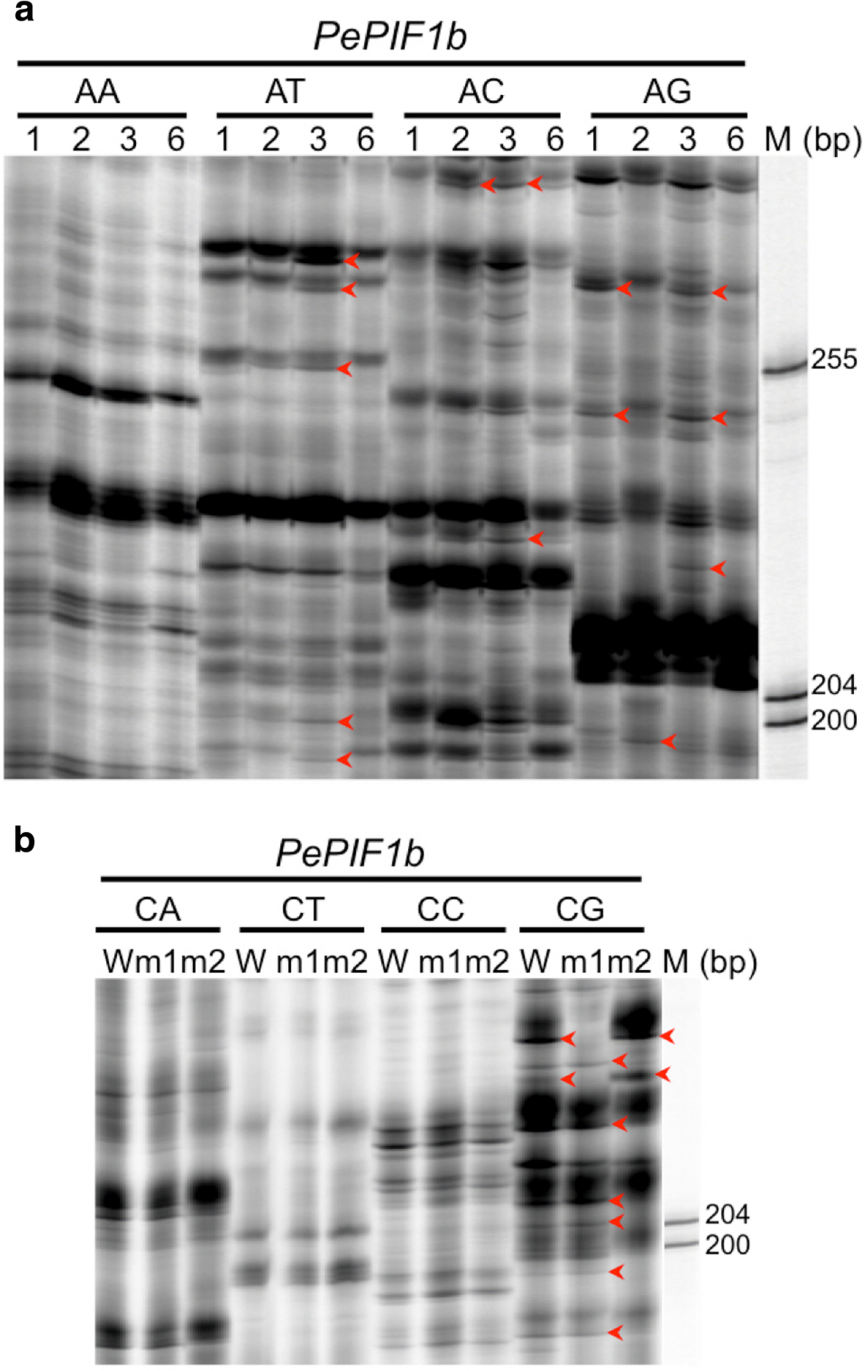
Transposon display profile of *PePIF1* in various PLB generations of KHM1219 (**a**) and the wild-type and somaclonal variants of KHM2180 with various pigmentation patterning (**b**). **a** Seedlings from various PLB generations of KHM1219 (N1, N2, N3 and N6) are labeled 1, 2, 3, and 6. **b** Wild type and somaclonal variants of KHM2180 are labeled W, m1, and m2. The DNA size markers are indicated as “M” on the right and left side of the gel. AA, AT, AC, AG, CA, CT, CC, and CG indicate the second selective primers used for PCR amplification. The red arrowheads indicate the differentially amplified fragments with a new band

To assess the transposition activity of *PePIF1*, transposon display analysis was adopted to analyze the layout of the *PePIF1* sequence in the genomic DNAs of seedlings derived from consecutive PLB generations of KHM1219 and KHM2180, the normal and crystal PLBs of KHM487 (Fig. 1a, b), and individual flowers of KHM2180 with distinct pigmentation patterning (Fig. 1e-h). For transposon display analysis, *PePIF1a*- and *PePIF1b*-specific primers were designed from the weak conserved sub-terminal regions with the addition of 2-bp selection primers (see Methods, Fig. 3). Most amplified fragments of *PePIF1a* and *PePIF1b* were identical for the consecutive generations of KHM2180 as well as the normal and crystal PLBs of KHM487 (Additional file 5: Figure S3). The amplification of *PePIF1b* revealed several differential bands in the consecutive generations of KHM1219 and the individual flowers of KHM2180 with distinct pigmentation patterning (Fig. 5, red arrowhead). Considering the two somaclonal variants (Fig. 1f, g) of KHM2180 were resulted from the mother plants (Fig. 1e) with independent mutation events, the differential bands were identified for the loss of *PePIF1b* insertion, which means most bands were found in WT and m1, but not in m2 for the loss of *PePIF1b* insertion (Fig. 5b). The results suggest that *PePIF1b* could be transposed and resulted in a change of amplified fragment patterns in the genome of KHM1219 during PLB micropropagation and also in the mutant flowers of KHM2180. Therefore, *PePIF1b* could be an active transposon in the *Phalaenopsis* genome. In contrast, the banding pattern for *PePIF1a* was not changed, thus excluding the possibility of contamination or genome rearrangement (Additional file 5: Figure S3).

The differentially amplified fragments (Fig. 5, red arrowhead) were recovered and sequenced to confirm the presence of *PePIF1* insertion, then mapped to the *P. equestris* genome to identify the genes inserted with or near *PePIF1* during consecutive generations of KHM1219 or the somaclonal variants of KHM2180. Among them, 27 genes were identified with *PePIF1* insertion, and only two, PEQU_19321 and PEQU_41063, were inserted in the coding sequences and the others were located in the intron regions (Additional file 6: Table S3). In addition, 52 predicted genes were found with 26 *PePIF1s* locating in the intergenic region, such as genes encoding DEMETER and arginine N-methyltransferase (Additional file 6: Table S3). We designed primers for five candidate insertion sites, PEQU_09928, PEQU_19321, PEQU_26837, PEQU_34751, and PEQU_41063, for PCR amplification of the flanking sequences to confirm the *PePIF1* insertions. PCR amplification with both gene-specific and *PePIF1b*-specific primers provided more precise result for the gene and TE structures than transposon display, which used both *PePIF1b*-specific primer and adaptor primer. PEQU_26837, encoding serine/threonine-protein kinase GRIK2-like protein [40], produced differentially amplified fragments in both KHM1219 and KHM2180 (Additional file 7: Figure S4a), where the amplified 3-kb fragments of PEQU_26837 were the same in m2 and wt of KHM2180, suggesting the situation for loss of insertions in the mutants. However, m1 contained a large fragment with 6-kb sequence, so the PCR amplification with gene specific primers revealed detail information that the lost 3-kb fragment in m1 became a new insertion with 6-kb sequence and that was not detected in transposon display analysis. Similar situation was also found in PEQU_19321, encoding U-box domain-containing protein 43-like protein [41], where the amplified DNA fragment was larger than 3-kb sequence, and was another copy lost in wild type but present in m1 and m2, but they were not detected in transposon display analysis (Additional file 7: Figure S4b). However, the amplified patterns for PEQU_41063, PEQU_09928, and PEQU_34751, encoding disease resistance protein RGA3, histone-lysine N-methyltransferase, and RNA demethylase ALKBH5-like protein, respectively, were not distinct among all the samples tested (Additional file 7: Figure S4c-e). These results suggest that there were false positives present within these differential bands in transposon display or DNA samples prepared for PCR testing did not include cells where transposition occurred.

## Discussion

### Identification of a putative active *PIF*-like TE from a *Phalaenopsis* genome sequence

In this study, we used a strategy combining the experimental analysis and in silico genome mining approaches and identified an active *PIF*-like TE, *PePIF1*, in a *Phalaenopsis* genome. First, candidate TEs were identified by their transcription activities of TPase during consecutive PLB micropropagation by using microarray assay. Second, *PePIF1* was identified as a potential autonomous TE because of the presence of TIRs accompanying the coding sequences of TPase and ORF1 in a *Phalaenopsis* genome. Third, the transposition activities of *PePIF1* were investigated in consecutive PLBs and single flowers with changed pigmentation patterns by using transposon display assay.

### *PIF*-like elements in *Phalaenopsis* showed distinct evolutionary lineage

Several *PIF*-like elements have been identified from plant genomes and grouped into separate evolution lineages. The phylogenetic analysis of the catalytic regions of TPases of *PIF*-like elements from rice and other plants resulted in four lineages: A1~A5, B, C, and D [33]. Carrot contains fewer than 10 copies of putative autonomous *PIF*-like elements and they were grouped into a new M lineage, which contains *DcMasterA* and *MtPIF1* [34]. In addition, nine putative autonomous *PIF*-like elements are present in *Lotus japonicus* and they were grouped into the A3 lineage [42]. For *M. truncatula*, 22 putative autonomous and 67 non-autonomous *PIF*-like elements were grouped into three lineages, A5, D and M, and two new lineages, A6 and E [35]. Recently, 240 nonredundant *PIF-*like TPase sequences were amplified from 21 species of Triticeae genera and grouped into four main lineages [37]. Among them, 156 cDNA fragments from 15 species showed transcriptional activities [32]. In this study, we identified 66 putative autonomous *PePIF1* elements and grouped them into a novel P lineage with two families, *PePIF1a* and *PePIF1b*. These results indicate that the evolution of *PIF*-like elements strikingly differed among various plant species for both the phylogenetic lineages and their copy numbers, so the types and numbers of TEs from each plant species must be investigated to understand their effects on genome evolution.

### Abundant *PePIF1* may result from transposition and abortive gap repair (AGR) processes

*PePIF1* belongs to class II DNA transposons, which use the cut-and-paste mechanism for transposition, but more than a thousand copies of *PePIF1* were identified from a *Phalaenopsis* genome, including 66 predicted autonomous and 1221 defective elements. Length distribution analysis suggested that direct deletion of the putative autonomous elements usually occurred for generation of the defective elements, although other types of mutations and rearrangements, including nested insertions to increase the TE length, were also observed [35]. Alternatively, the production of these high-copy-defective elements may be due to the AGR model, which suggests that new defective elements are usually derived from the DNA repair system to refill the double-strand breakage in the location after autonomous-element excision [43]. Therefore, one *PePIF1* might transpose to another location by a cut-and-paste strategy but produce a new error-prone copy in the original site by a DNA repair system. In *M. truncatula*, various copy numbers of five lineages of *PIF*-like elements were affected by the efficiency of the transpositional activity or the AGR process [35]. The AGR model explained the presence of many copies of *PePIF1*, especially the defective elements containing both TIRs without middle sequences, and the transposition frequency accompanying the AGR process could be high in the *Phalaenopsis* genome. In addition, while the defective elements inserted in the genome, mutations (point mutations, insertion and deletion) occurred and leaded to the loss of one TIR or certain part of the element.

Several defective copies with only one end of elements might be from the assembly limitation of the *P. equestris* genome, which was assembled only from short-reads, and the most repetitive sequences, TIRs, were left as gaps or Ns in the assembled sequence. Therefore, the whole genome sequences assembled with short read-based sequences were difficult for the estimation of TE copy numbers. The repetitive sequences might be assembled together due to their similarity in sequences, so the true copy numbers should be higher than what was predicted from genomic sequences. In addition, the TIRs, as the most similar region, might be assembled together and then lost from the original copies. So the number of defective copies without one or two TIRs might be over estimated.

### Relationship of *PePIF1* and its MITE-like *mPePIF1*

Class-II DNA transposons are considered the ancestors of certain groups of MITEs and activate the transposition of these MITEs [44]. For example, *mPing* is suggested to be activated by its autonomous class-II DNA transposons, *Ping* and *Pong*, on the basis of their sequence similarities of TIRs and experimental analysis [29, 45]. In addition, several *PIF*-like elements show TIR sequence similarities to those of MITE families, including *Heartbreaker* from maize [46], *Kiddo* from rice [47], and *Krak* from carrot [34]. Therefore, we considered that MITE-like *miniature PePIF1* (*mPePIF1*) was derived from *PePIF1* and contained similar TIRs, TSDs, and sub-terminal sequences as *PePIF1* but without the coding capacity.

In addition, most transposition activities of TEs occurred via MITE-like elements but not autonomous elements [28, 29]. The primers used for transposon display were designed for the sub-terminal regions of *PePIF1a* or *PePIF1b*, so the transposition activities of *PePIF1a* or *PePIF1b* could be distinguished. However, the sub-terminal sequences of *PePIF1a* or *PePIF1b* were similar to their derived MITE-like *mPePIF1*, so transposition of *PePIF1* could be produced by the MITE-like *mPePIF1*, derived from *PePIF1a* or *PePIF1b*.

Moreover, the transposon display result showed only *PePIF1b* containing the transposition activity, although the differential transcription activities among PLB generations were detected for *EFCP_7972*, which belongs to *PePIF1a*. Since *PePIF1a* and *PePIF1b* showed the same TIRs, it is plausible that *PePIF1b* was transposed by the expressed TPase of *PePIF1a*. As the situation for *mPing* and its autonomous class-II DNA transposons, *Ping* and *Pong*, they share the similar TIR sequences and thought to be responsible for *mPing* transposition [29, 45].

### *mPePIF1* derived from transposition and amplification of themselves

Although the transposition of MITEs was activated by other DNA transposons, MITEs have the potential to amplify to high copy numbers, whereas DNA transposons amplify to fewer than 50 copies [48]. For example, most rice cultivars contain fewer than 50 copies of *mPin*g, but the Gimbozu EG4 strain has more than 1000 copies amplified [48]. The amplification of *mPing* was explained by the DNA repair system for the double-strand breaks, with *mPing* excision by using the other *mPing* copy from the sister chromatid or homologous chromosome [49]. Therefore, most *mPePIF1s* had similar lengths of 200~300-bp sequences and were probably produced by the transposition and amplification of themselves but not by deletion or the AGR process of *PePIF1*.

### Genes with *PePIF1* insertions might cause the somaclonal variants of KHM2180 and KHM1219

The mutations with TE insertions and excision in gene coding regions may change protein functions and enzymatic activities [50], whereas the upstream regulatory regions with TE insertions may modify tissue-specific gene expression patterns [51]. With use of transposon display, the differentially amplified DNA fragments in the genomes of KHM1219 and KHM2180 were sequenced and found to possibly cause the deranged phenotypes of the somaclonal variants. In all, 27 genes were identified with *PePIF1* insertions in the gene regions, with 26 *PePIF1s* locating in the intergenic region between 52 genes. Among them, histone-lysine N-methyltransferase [52] and DEMETER [53] may play roles in epigenetic regulation of the somaclonal variants during micropropagation in *Phalaenopsis*, as was previously found for whole-genome hypomethylation in somaclonal mantled palms [13] and *Phalaenopsis* [54]. In addition, polyadenylate-binding proteins for binding to poly(A) RNA [55], RNA demethylase [56], and zinc finger CCCH domain-containing protein for pre-mRNA splicing [57] are responsible for the RNA process at a post-transcriptional level and may also play roles in the somaclonal variations in *Phalaenopsis*.

However, *PePIF1* was the first identified active transposon in *Phalaenopsis*, and other TEs or factors such as DNA methylation may also affect the somaclonal variants. Therefore, our results show the predicted genes with *PePIF1* insertions in KHM1219 and KHM2180, but more evidence is needed for elucidating their roles in somaclonal variations during micropropagation.

With the amplified and sequenced differential DNA fragments of *PePIF1* in the genomes of normal and somaclonal variants of KHM1219 and KHM2180, we thought that *PePIF1* was an active transposon in a *Phalaenopsis* genome. The various generations of PLBs from KHM1219 as well as the normal and somaclonal variants of KHM2180 were induced from individual plants of *P.* Sogo Berry ‘KHM1219’ and *P.* I-Hsin The Big Bang ‘KHM2180,’ respectively, so there were no pre-existing genomic DNA polymorphisms in the analyzed samples before the experiments. However, mitotic recombination is also a major cause of the polymorphic insertion of MITE elements, which is also possible for the differentially amplified fragments of *PePIF1* [58].

## Conclusions

In this study, a strategy combining experimental analysis and in silico genome mining was used for successful identification of an active *PIF*-like TE, *PePIF1*, in a *Phalaenopsis* genome. The identification of *PePIF1* can provide more understanding of the *Phalaenopsis* genome structure and somaclonal variations during micropropagation, which would benefit orchid breeding and production.

## Methods

### Plant materials

Three pairs of normal and somaclonal variants of *Phalaenopsis* were used in this study, including *P.* Brother Spring Dancer ‘KHM487’ (Fig. 1a, b), *P.* Sogo Berry ‘KHM1219’ (Fig. 1c, d), and *P.* I-Hsin The Big Bang ‘KHM2180’ (Fig. 1e-h), abbreviated to KHM487, KHM1219, and KHM2180, respectively. These somaclonal variants contain various phenotypes, including KHM487 with crystal-like PLBs, which leads to peloric flowers in mature plants (Fig. 1b). KHM1219 has peloric flowers (Fig. 1d), and KHM2180 shows distinct flower pigmentation patterning (Fig. 1e-h). Two individual plants of KHM1219 and KHM2180 were chosen and used for inducing callus to grow into PLBs. These PLBs were used for further inducing callus and for micropropagation of consecutive generations of PLBs, and grown into seedlings, then whole plants. Pooled seedlings of PLB in the same generations of KHM1219 and KHM2180 were used for quantitative real-time PCR and transposon display analyses. Single flowers of mother plants (Fig. 1e) and somaclonal variants (Fig. 1f, g) of KHM2180 were analyzed by transposon display. All plants were provided by I-Hsin Biotechnology Inc. (Chiayi, Taiwan) and kept under natural light and controlled temperature from 23 °C to 27 °C in the greenhouse at National Cheng Kung University (Tainan, Taiwan).

### *Phalaenopsis* transcriptome microarray

The Agilent custom Orchid Oligo array (4 × 44 K) was designed for 14,732 unigenes chosen among the 84,617 unigenes in OrchidBase [4, 38]. RNA samples extracted from various generations of *Phalaenopsis* tissue culture derived from KHM487 were used for analysis of differential expression profiles following the Agilent eArray 5.0 program with the manufacturer’s recommendations [38].

### Quantitative real-time PCR

For RNA extraction, the PLBs of various generations or stage-3 floral buds were immersed in liquid nitrogen and stored at − 80 °C. Total RNA extraction, cDNA synthesis, and quantitative real-time PCR were as described [59]. For each TE candidate, primer pairs within the gene-specific regions were designed and are listed in Additional file 8: Table S4. After amplification, melting curve analysis was used to verify amplicon specificity and primer dimer formation. A housekeeping gene, PeActin4 (PACT4, AY134752), displayed expression stability for M = 1.02 in GeNorm analysis [60] and was used for normalization [59, 61–64]. Data are mean ± SEM calculated from triplicate data, and experiments involved three independent biological repeats.

### Investigation of the genome structures of TE candidates

The predicted TE candidates were first mapped to the whole-genome sequence of *P. equestris* by using a BLASTN algorithm with a cutoff E value of 1.0 × 10^− 10^. The flanking regions of 10-kb sequences of these predicted TEs were used to screen for the presence of terminal repeats by using a BLAST2 algorithm with more than 11-bp matches. Then the identified repeats were verified for their co-localization with the predicted TEs in the other regions of the *P. equestris* genome. The target-site duplications (TSDs) were identified from the flanking sequences of the terminal inverted repeats (TIRs).

### Phylogenetic analysis of *PIF*-like TPases

The amino acids of the catalytic DDE domain of TPase for each *PIF*- and *Pong*-like TE were corrected for the frameshifts caused by 1–2 bp insertions or deletions and used to construct the phylogenetic tree with the maximum likelihood method by using ClustalW [65] and MUSCLE [66] implemented in MEGA v6 [67]. The 1000 bootstrapping datasets were used to estimate the confidence for each tree clade. Sequence data are available at NCBI with the accession numbers for *PePIF1a* and *PePIF1b* of MG470826 and MG470827, respectively.

### Copy number estimation of *PIF*-like elements

The sequences for TIRs and coding sequences for ORF1 and TPase were mapped to the whole-genome sequence of *P. equestris* to screen for other copies of *PePIF1* by using BLASTN and TBLASTX algorithms for TIR and coding sequences, respectively, with a cutoff E value of 1.0 × 10^− 10^. The predicted autonomous elements were identified by the presence of the coding sequences for TPase and ORF1 between two TIRs with maximum length of 30-kb sequences. All these elements of *PePIF1* were mapped to the predicted proteins from the genomic sequences of *P. equestris* to identify the predicted proteins with *PePIF1* insertions.

### Transposon display for the transposition activity of *PePIF1*

The transposon display procedure was modified from a previous report [46]. DNA samples were digested with *Bfa*I and ligated with adapters. The pre-selective amplifications involved use of an adapter-complementary primer and another TE-specific primer, PePIF1a_TD1 or PePIF1b_TD1, complementary to the weak conserved sub-terminal sequence of *PePIF1* (Additional file 8: Table S4) with the temperature cycling parameters 94 °C for 5 min; 24 cycles of 94 °C for 30 s, 59 °C for 30 s, and 72 °C for 1 min, and a final cycle of 72 °C for 5 min. Selective amplification for detection involved use of 1/4-diluted pre-selective amplification products, adapter-complementary primer+NN and IRDye-700-labeled TE-specific PePIF1a_TD2 or PePIF1b_TD2 (Additional file 8: Table S4) (Protech Technology Enterprise Co., Taipei) with a “touchdown” protocol: 94 °C for 5 min; 35 cycles of 94 °C for 30 s, 69–61 °C for 30 s, and 72 °C for 1 min, and a final cycle of 72 °C for 5 min. The annealing temperature was reduced from 69 °C to 61 °C in 1 °C increments for each cycle. Separation of these fluorescently labeled transposon display fragments was performed with 7.5% polyacrylamide sequencing gel, with imaging by use of the Li-COR 4300 DNA Analyzer System (Li-COR Biotechnology, Lincoln, NB, USA). The differentially amplified fragments were recovered from the gel, cloned into the T-easy vector (Invitrogen), and randomly sequenced for 6–8 clones.

## Additional files

Additional file 1: **Figure S1.** Transcription activity of *EICPS_047* during tissue culture of KHM1219 (a) and KHM2180 (b). Data are mean ± SEM from experiments performed in triplicate. (DOCX 110 kb)

Additional file 2: **Table S1.** Mapping of the three transposable element candidates to the whole-genome sequence of *Phalaenopsis equestris*. (DOCX 13 kb)

Additional file 3: **Figure S2.** Multiple alignment of the amino acid sequence of ORF1s (a) and TPases (b). (DOCX 778 kb)

Additional file 4: **Table S2.** Predicted autonomous elements of *PePIF1* in the genome sequence of *P. equestris*. (DOCX 21 kb)

Additional file 5: **Figure S3.** Transposon display profile of *PePIF1* in normal and crystal-like PLBs of KHM487 (a) and various PLB generations of KHM2180 (b). (DOCX 301 kb)

Additional file 6: **Table S3.** The insertion sites of *PePIF1* in the whole-genome sequence of *P. equestris (DOCX 18 kb)*

Additional file 7: **Figure S4.** Confirmation of the transposon display result by PCR with the primers designed in the flanking sequences of five candidate insertion sites in various PLB generations of KHM1219 and the wild type and somaclonal variants of KHM2180 (DOCX 239 kb)

Additional file 8: **Table S4.** Primers used in this study. (DOCX 13 kb)

## Abbreviations

AGR: Abortive gap repair
MITEs: Miniature inverted-repeat transposable elements
mPIF: *Miniature P Instability Factor*
ORF: Open reading frame
PIF: *P Instability Factor*
PLBs: Protocorm-like bodies
TEs: Transposable elements
TIRs: Terminal inverted repeats
TPase: Transposase
TSDs: Target-site duplications
