# Supplementary material for: PePIF1, a P-lineage of PIF-like transposable element identified in protocorm-like bodies of Phalaenopsis orchids
Source: BMC Genomics. 2019 Jan 9;20:25. doi: 10.1186/s12864-018-5420-4 (PMC6327408; doi:10.1186/s12864-018-5420-4)
Supplement: Supplementary file 1 — Figure S1. Transcription activity of EICPS_047 during tissue culture of KHM1219 (a) and KHM2180 (b). Data are mean ± SEM from experiments performed in triplicate. (DOCX 110 kb) [file 12864_2018_5420_MOESM1_ESM.docx]

**
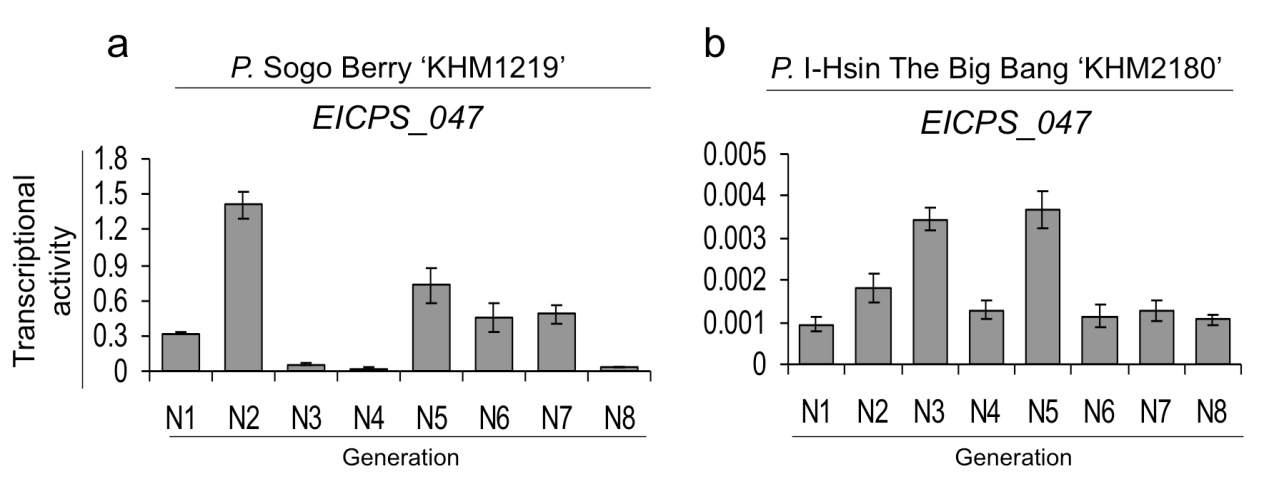
**

**Additional file 1: Figure S1.** Transcription activity of *EICPS_047* during tissue culture of KHM1219 (a) and KHM2180 (b). Data are mean±SEM from experiments performed in triplicate.
