# Supplementary material for: PePIF1, a P-lineage of PIF-like transposable element identified in protocorm-like bodies of Phalaenopsis orchids
Source: BMC Genomics. 2019 Jan 9;20:25. doi: 10.1186/s12864-018-5420-4 (PMC6327408; doi:10.1186/s12864-018-5420-4)
Supplement: Supplementary file 2 — Table S1. Mapping of the three transposable element candidates to the whole-genome sequence of Phalaenopsis equestris. (DOCX 13 kb) [file 12864_2018_5420_MOESM2_ESM.docx]

**Additional file 2: Table S1.** Mapping of the three transposable element candidates to the whole-genome sequence of *Phalaenopsis equestris*.

| Name in OrchidBase and Orchid Oligo Array | Length (bp) | No. of hits | Location of Top hit  in *Phalaenopsis* genome |
| --- | --- | --- | --- |
| *EICPS_047* | 750 | 1 | Scaffold000002: 24521329-24522103 |
| *EFCP_7972* | 747 | 369 | Scaffold000759: 211546-211981 |
