# Supplementary material for: PePIF1, a P-lineage of PIF-like transposable element identified in protocorm-like bodies of Phalaenopsis orchids
Source: BMC Genomics. 2019 Jan 9;20:25. doi: 10.1186/s12864-018-5420-4 (PMC6327408; doi:10.1186/s12864-018-5420-4)
Supplement: Supplementary file 3 — Figure S2. Multiple alignment of the amino acid sequence of ORF1s (a) and TPases (b). (DOCX 778 kb) [file 12864_2018_5420_MOESM3_ESM.docx]

**
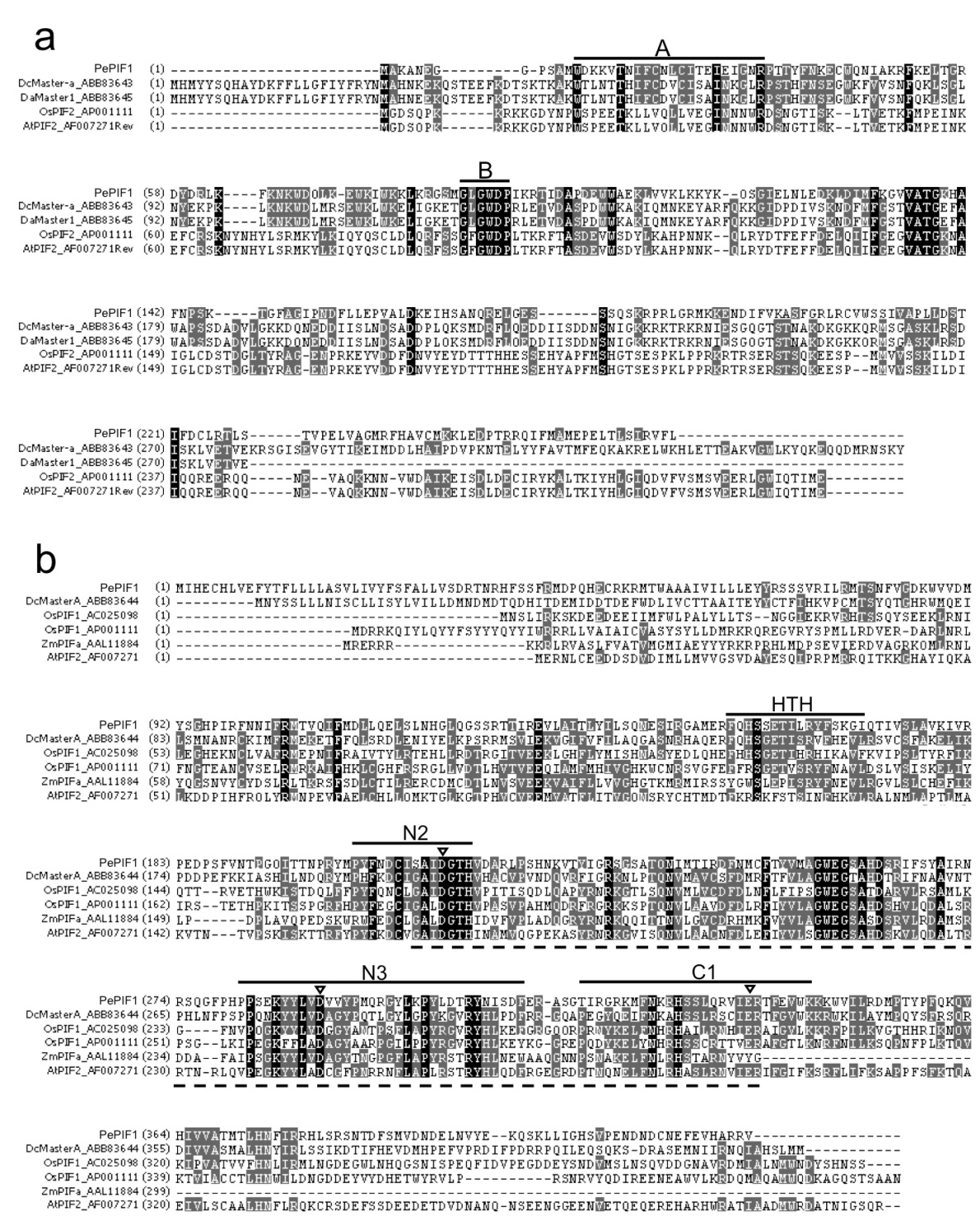
**

**Additional file 3: Figure S2.** Multiple alignment of the amino acid sequence of ORF1s (a) and TPases (b). (a) Horizontal lines represent the conserved blocks A and B. (b) Triangles indicate the DDE motif within the three conserved blocks (N2, N3 and C1) that comprise the catalytic domain and are represented by horizontal lines. Another horizontal line indicates the predicted helix-turn-helix (HTH) domain. The dashed line indicates the catalytic DDE region for phylogenetic analysis. The other *PIF*-like elements were named according to the species initials followed by their GenBank accession number.
