# Supplementary material for: PePIF1, a P-lineage of PIF-like transposable element identified in protocorm-like bodies of Phalaenopsis orchids
Source: BMC Genomics. 2019 Jan 9;20:25. doi: 10.1186/s12864-018-5420-4 (PMC6327408; doi:10.1186/s12864-018-5420-4)
Supplement: Supplementary file 4 — Table S2. Predicted autonomous elements of PePIF1 in the genome sequence of P. equestris. (DOCX 21 kb) [file 12864_2018_5420_MOESM4_ESM.docx]

**Additional file 4: Table S2.** Predicted autonomous elements of *PePIF1* in the genome sequence of *P. equestris*.

| Location in scaffold sequence (bp) | | | Length  (bp) | ORF1 location | TPase location | Direction |
| --- | --- | --- | --- | --- | --- | --- |
| ***PePIF1a* family** | | | | | | |
| Scaffold000002 | : | 31977014-31891730 | 4716 | 31978871-31979372 | 31979658-31980948 | + |
| Scaffold000002 | : | 76810461-76815842 | 5381 | 76812223-76812992 | 76813454-76815244 | + |
| Scaffold000003 | : | 328915-333716 | 4801 | 331349-332085 | 329558-330856 | - |
| Scaffold000027 | : | 124052-131826 | 7774 | 129169-130036 | 130575-131562 | + |
| Scaffold000058 | : | 153793-158794 | 5001 | 155687-156358 | 156863-158233 | + |
| Scaffold000125 | : | 996322-1001925 | 5603 | 997863-999553 | 1000034-1001355 | + |
| Scaffold000126 | : | 145024-173481 | 28457 | 161930-162256 | 163076-164354 | + |
| Scaffold000150 | : | 334511-338939 | 4428 | 336225-337063 | 337568-338572 | + |
| Scaffold000153 | : | 85477-91213 | 5736 | 87501-88238 | 88565-89973 | + |
| Scaffold000224 | : | 3277012-3289473 | 12461 | 3286196-3286728 | 3287329-3288628 | + |
| Scaffold000224 | : | 6135531-6141139 | 5608 | 6138045-6138769 | 6139226-6140405 | + |
| Scaffold000504 | : | 809991-815462 | 5471 | 812174-812907 | 813287-814608 | + |
| Scaffold000531 | : | 920316-926078 | 5762 | 922885-923587 | 923998-925287 | + |
| Scaffold000568 | : | 185247-189779 | 4532 | 187586-188140 | 185609-186984 | - |
| Scaffold000740 | : | 134979-139707 | 4728 | 136933-137609 | 137851-138923 | + |
| Scaffold000759 | : | 210511-215421 | 4910 | 213062-213784 | 211253-212598 | - |
| Scaffold000840 | : | 5761487-5766644 | 5157 | 5763383-5764060 | 5764545-5765894 | + |
| Scaffold000841 | : | 24859-29782 | 4923 | 27381-27923 | 25428-26766 | - |
| Scaffold000866 | : | 82086-87138 | 5052 | 83876-84420 | 85029-86350 | + |
| Scaffold001013 | : | 1189846-1194793 | 4947 | 1191758-1192491 | 1192940-1194216 | + |
| Scaffold001131 | : | 24547-29637 | 5090 | 26930-27558 | 25230-26496 | - |
| Scaffold001143 | : | 153526-158473 | 4947 | 155232-155975 | 156416-157759 | + |
| Scaffold001307 | : | 755962-761135 | 5173 | 757947-758671 | 759105-760420 | + |
| Scaffold198769 | : | 3064-7818 | 4754 | 5534-6220 | 3630-4970 | - |
| Scaffold210921 | : | 57262-63412 | 6150 | 60178-60912 | 61393-62803 | + |
| Scaffold219150 | : | 185150-198969 | 13819 | 187663-188360 | 185953-187191 | - |
| Scaffold227736 | : | 48008-52992 | 4984 | 49984-50506 | 51097-52511 | + |
| Scaffold229743 | : | 35521-49791 | 14270 | 38424-38564 | 36128-37489 | - |
| Scaffold233353 | : | 7610-12762 | 5152 | 9496-10110 | 10732-12136 | + |
| ***PePIF1b family*** | | | | | | |
| Scaffold000002 | : | 18151538-18168430 | 16892 | 18161946-18162786 | 18163085-18164236 | + |
| Scaffold000002 | : | 48739394-48744275 | 4881 | 48742051-48742792 | 48740385-48741613 | - |
| Scaffold000002 | : | 61613715-61620107 | 6392 | 61618339-61619210 | 61616777-61618266 | - |
| Scaffold000002 | : | 72118390-72123194 | 4804 | 72120706-72121412 | 72118913-72120262 | - |
| Scaffold000022 | : | 284340-293874 | 9534 | 290842-291631 | 289111-290608 | - |
| Scaffold000022 | : | 708114-714694 | 6580 | 709310-710117 | 710281-711725 | + |
| Scaffold000050 | : | 185540-190779 | 5239 | 188274-189135 | 186599-187777 | - |
| Scaffold000090 | : | 945043-951294 | 6251 | 946719-947589 | 947867-949196 | + |
| Scaffold000098 | : | 1314552-1323118 | 8566 | 1320271-1321061 | 1318444-1319922 | - |
| Scaffold000123 | : | 547492-553548 | 6056 | 551282-552119 | 549696-550971 | - |
| Scaffold000224 | : | 5656430-5663245 | 6815 | 5661220-5661697 | 5658520-5660861 | - |
| Scaffold000261 | : | 47499-56788 | 9289 | 49151-49965 | 50376-51729 | + |
| Scaffold000318 | : | 91634-99445 | 7811 | 96978-97722 | 95223-96510 | - |
| Scaffold000420 | : | 78976-86277 | 7301 | 82710-83339 | 83788-85083 | + |
| Scaffold000420 | : | 91912-97848 | 5936 | 94160-94905 | 95329-96647 | + |
| Scaffold000468 | : | 1227186-1234085 | 6899 | 1228639-1229351 | 1229763-1231110 | + |
| Scaffold000495 | : | 36831-43239 | 6408 | 38284-39004 | 39426-40783 | + |
| Scaffold000540 | : | 1947157-1954490 | 7333 | 1952526-1953401 | 1950884-1952246 | - |
| Scaffold000654 | : | 75825-81338 | 5513 | 78352-79084 | 76503-77939 | - |
| Scaffold000716 | : | 77632-85905 | 8273 | 80424-81299 | 81533-82986 | + |
| Scaffold000746 | : | 31107-39659 | 8552 | 36910-37588 | 35098-36357 | - |
| Scaffold000873 | : | 1689699-1696512 | 6813 | 1694508-1695129 | 1692727-1694176 | - |
| Scaffold000926 | : | 80592-87693 | 7101 | 85471-86292 | 83816-85157 | - |
| Scaffold000949 | : | 633157-655950 | 22793 | 648170-648723 | 649645-650780 | + |
| Scaffold001019 | : | 271441-276523 | 5082 | 272750-273610 | 273867-275183 | + |
| Scaffold001051 | : | 48246-55290 | 7044 | 50116-50980 | 51288-52893 | + |
| Scaffold001105 | : | 48422-57428 | 9006 | 50145-50538 | 51044-52602 | + |
| Scaffold001146 | : | 233143-240459 | 7316 | 234891-235202 | 235827-237314 | + |
| Scaffold001210 | : | 129864-139806 | 9942 | 132557-133102 | 130559-131919 | - |
| Scaffold199124 | : | 18529-25251 | 6722 | 19623-20341 | 20784-22122 | + |
| Scaffold207964 | : | 12439-19681 | 7242 | 14007-14811 | 15040-16397 | + |
| Scaffold212195 | : | 144013-149731 | 5718 | 146406-147120 | 147611-148931 | + |
| Scaffold217426 | : | 238593-245499 | 6906 | 242870-243778 | 241228-242472 | - |
| Scaffold223895 | : | 19287-24986 | 5699 | 22195-22911 | 20552-21693 | - |
| Scaffold232289 | : | 39652-47025 | 7373 | 44716-45464 | 42926-44237 | - |
| Scaffold234354 | : | 2887-8789 | 5902 | 5345-6012 | 6467-8055 | + |
| Scaffold234829 | : | 78224-86118 | 7894 | 82782-84434 | 81594-82762 | - |
