# Supplementary material for: PePIF1, a P-lineage of PIF-like transposable element identified in protocorm-like bodies of Phalaenopsis orchids
Source: BMC Genomics. 2019 Jan 9;20:25. doi: 10.1186/s12864-018-5420-4 (PMC6327408; doi:10.1186/s12864-018-5420-4)
Supplement: Supplementary file 5 — Figure S3. Transposon display profile of PePIF1 in normal and crystal-like PLBs of KHM487 (a) and various PLB generations of KHM2180 (b). (DOCX 301 kb) [file 12864_2018_5420_MOESM5_ESM.docx]

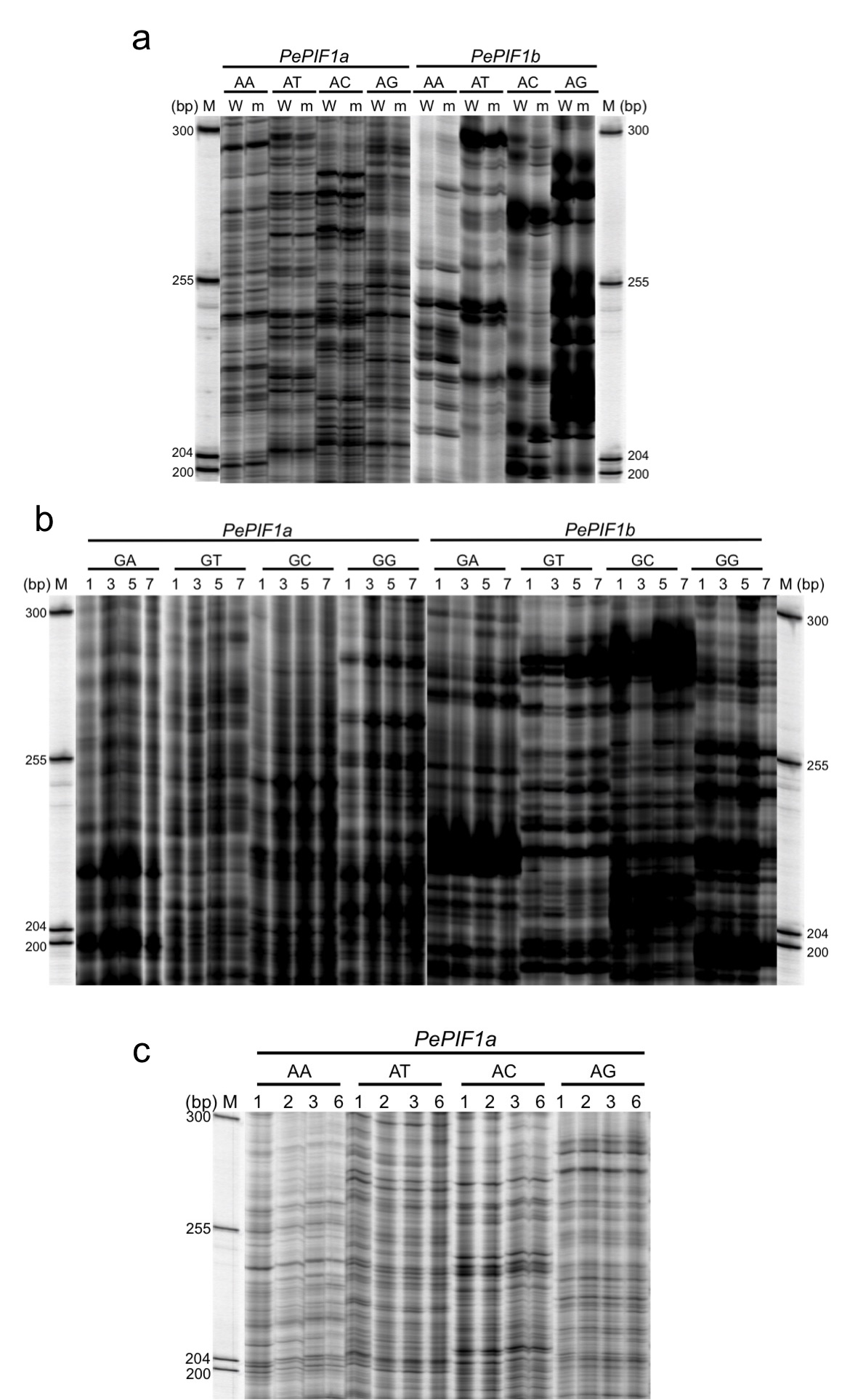


**Additional file 5: Figure S3.** Transposon display profile of *PePIF1* in normal and crystal-like PLBs of KHM487 (a) and various PLB generations of KHM2180 (b) and KHM1219 (c). (a) "W" and "m" indicate normal type and crystal-like PLBs of KHM487, respectively. (b) Seedlings from various PLB generations of KHM2180 (N1, N3, N5 and N7) are labeled 1, 3, 5, and 7. (c) Seedlings from various PLB generations of KHM1219 (N1, N2, N3 and N6) are labeled 1, 2, 3, and 6. The DNA size markers are indicated "M" on the right and left side of the gel. AA, AT, AC, AG, GA, GT, GC, and GG indicate the second selective primers used for PCR amplification.
