# Supplementary material for: PePIF1, a P-lineage of PIF-like transposable element identified in protocorm-like bodies of Phalaenopsis orchids
Source: BMC Genomics. 2019 Jan 9;20:25. doi: 10.1186/s12864-018-5420-4 (PMC6327408; doi:10.1186/s12864-018-5420-4)
Supplement: Supplementary file 6 — Table S3. The insertion sites of PePIF1 in the whole-genome sequence of P. equestris (DOCX 18 kb) [file 12864_2018_5420_MOESM6_ESM.docx]

**Additional file 6: Table S3.** The insertion sites of *PePIF1* in the whole-genome sequence of *P. equestris.*

| Gene name | Predicted protein annotation | Gene name | Predicted protein annotation |
| --- | --- | --- | --- |
| Predicted cds genes with PePIF1 insertion in KHM1219 | | | |
| PEQU_00620 | suppressor of mec-8 and unc-52 protein homolog 1 | PEQU_18078 | 60S ribosomal protein L36-3-like protein |
| PEQU_01883 | universal stress protein PHOS34-like protein | PEQU_18553 | pentatricopeptide repeat-containing protein |
| PEQU_06426 | ubiquitin-conjugating enzyme 15-like protein | PEQU_26837 | serine/threonine-protein kinase GRIK2-like protein |
| PEQU_09928 | histone-lysine N-methyltransferase | PEQU_32234 | uncharacterized protein |
| PEQU_10184 | kinase-interacting protein 1 | PEQU_34751 | RNA demethylase ALKBH5-like protein |
| PEQU_13380 | uncharacterized protein | PEQU_36744 | leucine-rich repeat receptor-like serine/threonine-protein kinase |
| PEQU_14493 | YbaK/aminoacyl-tRNA synthetase-associated domain isoform 1 | PEQU_38422 | thiosulfate sulfurtransferase 16 |
| PEQU_14869 | GTP-binding protein | PEQU_39524 | GTPase activating protein 1-like protein |
| PEQU_16059 | copper-transporting ATPase PAA1 | PEQU_41256 | putative nuclease HARBI1 |
| PEQU_16547 | polyadenylate-binding protein-interacting protein 11-like protein |  |  |
| Predicted cds genes with PePIF1 nearby in KHM1219 | | | |
| PEQU_00766 | Pol polyprotein | PEQU_27455 | heavy metal-associated isoprenylated plant protein 32-like protein |
| PEQU_00767 | IGR protein motif | PEQU_27456 | F-box protein |
| PEQU_00859 | Retrovirus-related Pol polyprotein from transposon 297 family | PEQU_28235 | putative respiratory burst oxidase homolog protein H isoform X1 |
| PEQU_00860 | filament-like plant protein 3 | PEQU_28236 | Reverse transcriptase zinc-binding domain |
| PEQU_01800 | zinc finger CCCH domain-containing protein 27 | PEQU_28762 | UDP-glucose 4-epimerase GalE |
| PEQU_01801 | ethylene response protein | PEQU_28763 | uncharacterized protein |
| PEQU_06891 | UDP-glucose--hexose-1-phosphate uridylyltransferase | PEQU_28887 | probable protein phosphatase |
| PEQU_06892 | probable serine acetyltransferase 1 | PEQU_28888 | rolling stone-like protein |
| PEQU_08341 | uncharacterized protein | PEQU_30705 | RVT_1 domain-containing protein |
| PEQU_08342 | Retrovirus-related Pol polyprotein from transposon TNT 1-94 | PEQU_30706 | uncharacterized protein |
| PEQU_10026 | soluble starch synthase 2-2 | PEQU_33095 | Reverse transcriptase zinc-binding domain |
| PEQU_10027 | rve domain-containing protein | PEQU_33096 | probable protein S-acyltransferase 7 |
| PEQU_10725 | Calcineurin-like metallo-phosphoesterase superfamily protein | PEQU_34012 | purple acid phosphatase 17-like |
| PEQU_10726 | arginine N-methyltransferase | PEQU_34013 | tRNA (guanine-N(7)-)-methyltransferase non-catalytic subunit WDR4 isoform X2 |
| PEQU_23551 | serine/threonine-protein kinase roco5 | PEQU_35130 | uncharacterized protein |
| PEQU_23552 | serotonin N-acetyltransferase 1 | PEQU_35131 | No significant similarity found |
| Predicted cds genes with PePIF1 insertion in KHM2180 | | | |
| PEQU_10852 | protein FREE1-like isoform X1 | PEQU_19337 | pre-mRNA-splicing factor ATP-dependent RNA helicase DEAH9 isoform X1 |
| PEQU_11706 | aconitate hydratase | PEQU_19321 | U-box domain-containing protein 43-like protein |
| PEQU_12813 | phosphatidylinositol glycan anchor biosynthesis class U protein | PEQU_37280 | retrotransposon protein |
| PEQU_17924 | aldehyde dehydrogenase family 3 member F1-like protein | PEQU_41063 | disease resistance protein RGA3 |
| Predicted cds genes with PePIF1 nearby in KHM2180 | | | |
| PEQU_00257 | hypothetical protein | PEQU_18075 | very-long-chain enoyl-CoA reductase-like protein |
| PEQU_00258 | serine-tRNA ligase | PEQU_18076 | E3 ubiquitin-protein ligase XB3-like protein |
| PEQU_04563 | lysine-tRNA ligase | PEQU_19503 | olfactory receptor 4S2 |
| PEQU_04564 | remorin-like protein | PEQU_19504 | transcriptional activator DEMETER-like isoform X2 |
| PEQU_06103 | putative nuclease HARBI1 | PEQU_21340 | uncharacterized protein |
| PEQU_06104 | thiamine pyrophosphokinase 1-like isoform X1 | PEQU_21341 | retrotransposon protein, putative, Ty1-copia sub-class |
| PEQU_14167 | xanthine dehydrogenase-like protein | PEQU_22796 | UBN2_3 domain-containing protein |
| PEQU_14170 | putative nuclease HARBI1 | PEQU_22797 | receptor-like protein kinase HSL1 |
| PEQU_14276 | E3 SUMO-protein ligase | PEQU_34291 | cationic peroxidase 1-like protein |
| PEQU_14279 | IQ-DOMAIN 1-like protein | PEQU_34292 | alanine-glyoxylate aminotransferase 2 homolog 3 |
