# Supplementary material for: PePIF1, a P-lineage of PIF-like transposable element identified in protocorm-like bodies of Phalaenopsis orchids
Source: BMC Genomics. 2019 Jan 9;20:25. doi: 10.1186/s12864-018-5420-4 (PMC6327408; doi:10.1186/s12864-018-5420-4)
Supplement: Supplementary file 7 — Figure S4. Confirmation of the transposon display result by PCR with the primers designed in the flanking sequences of five candidate insertion sites in various PLB generations of KHM1219 and the wild type and somaclonal variants of KHM2180 (DOCX 239 kb) [file 12864_2018_5420_MOESM7_ESM.docx]

**
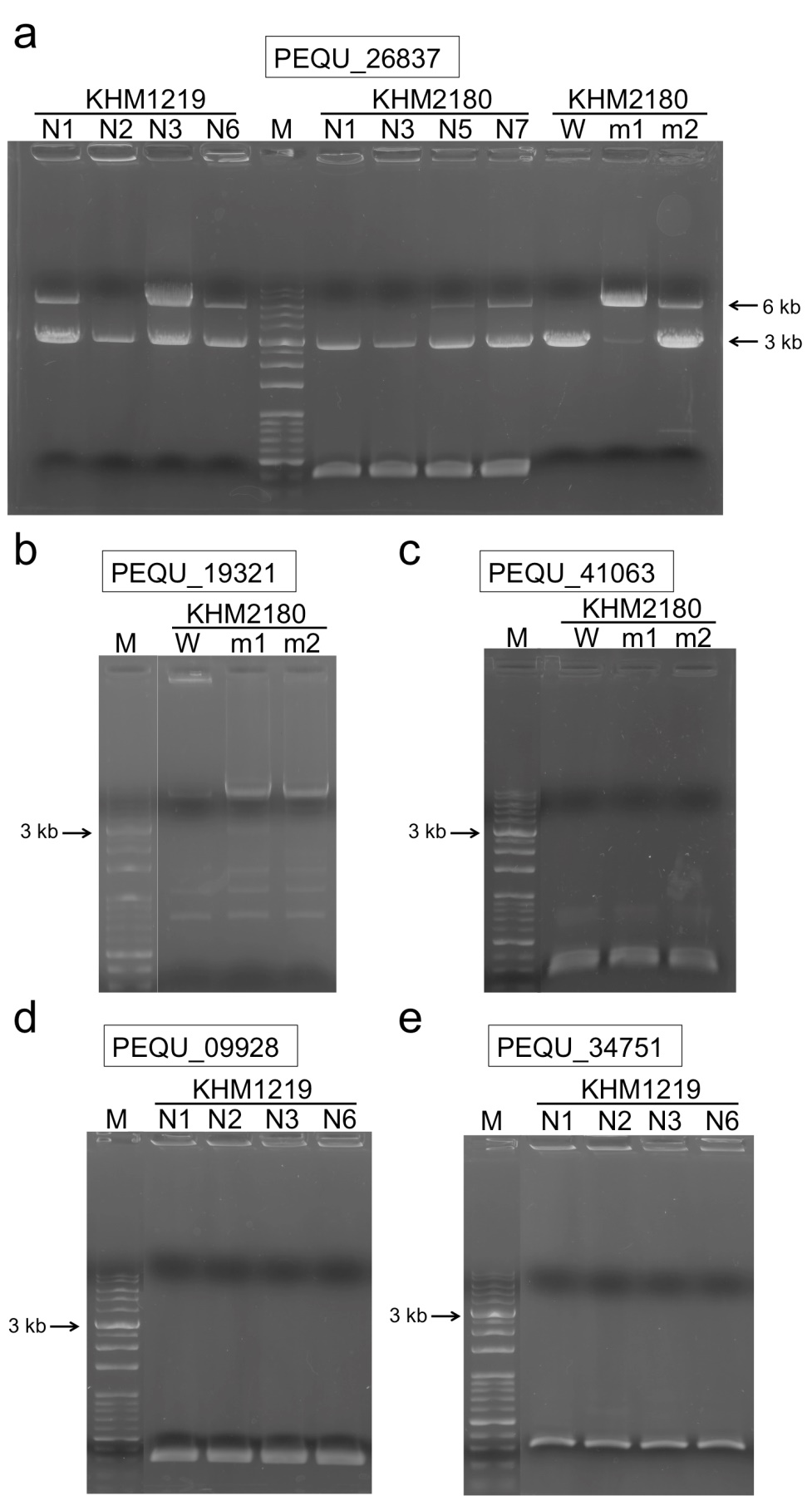
**

**Additional file 7: Figure S4.** Confirmation of the transposon display result by PCR approach with the primers designed in the flanking sequences of five candidate insertion sites, PEQU_26837 (a), PEQU_19321 (b), PEQU_41063 (c), PEQU_09928 (d), and PEQU_34751 (e) in various PLB generations of KHM1219 and the wild-type and somaclonal variants of KHM2180. (a-c) Wild type and somaclonal variants of KHM2180 are labeled W, m1, and m2. The DNA size markers are indicated as "M" on the right and left side of the gel.
