# Supplementary material for: PePIF1, a P-lineage of PIF-like transposable element identified in protocorm-like bodies of Phalaenopsis orchids
Source: BMC Genomics. 2019 Jan 9;20:25. doi: 10.1186/s12864-018-5420-4 (PMC6327408; doi:10.1186/s12864-018-5420-4)
Supplement: Supplementary file 8 — Table S4. Primers used in this study. (DOCX 13 kb) [file 12864_2018_5420_MOESM8_ESM.docx]

**Additional file 8: Table S4.** Primers used in this study.

| Primer name | Sequence |
| --- | --- |
| EICPS_047_realF | AGACACGAGGATATTATGTGGGAGATG |
| EICPS_047_realR | TGCAATGCGATCTCTCAATGCC |
| EFCP_7972_realF | TGGATACCCGATGCAGAGGGAC |
| EFCP_7972_realR | TGCCACGAATATTGCCACTTGC |
| PePIF1a_TD1 | TGTCATTTATACAACCATAACTACCAATAAGCTATTCTG |
| PePIF1a_TD2 | AATAAGCTATTCTGAATAAGCTGTCCCCAAAC |
| PePIF1b_TD1 | TACAAAACGCACTAACCAAACAGCTAAACAAC |
| PePIF1b_TD2 | AACAGCTAAACAACTTATTCAGAACAGGGGC |
